# The impact of teachers’ autonomy support on students’ academic performance from the perspective of hyperscanning: the mediating role of autonomy need satisfaction

**DOI:** 10.1093/scan/nsaf115

**Published:** 2025-10-31

**Authors:** Guoxia Wang, Qin Zhang, Lumeng Wang, Yi Liu

**Affiliations:** School of Psychology, Northeast Normal University, Changchun, China; School of Psychology, Northeast Normal University, Changchun, China; School Counseling Center, Shenzhen Bao’an District Hangxing School, Shenzhen, China; School of Psychology, Northeast Normal University, Changchun, China; School of Psychology, Northeast Normal University, Changchun, China

**Keywords:** fNIRS, hyperscanning, interpersonal neural synchronization, teacher autonomy support, autonomy need satisfaction

## Abstract

Previous research on self-determination theory has primarily focused on analyzing experiences and behaviors, without fully elucidating the neural basis of how teacher autonomy support influences students’ academic performance. In this study, four individuals were selected to act as teachers, while 42 individuals were assigned as students. The study manipulated teacher autonomy support and control styles. By simulating the real teaching process, functional near-infrared spectroscopy (fNIRS) hyperscanning technology was used to examine how teachers’ autonomy support style affected students’ autonomous motivation, academic emotions, and test scores. The behavioral findings indicated that, in comparison to the teachers’ control style, students exposed to the teacher autonomy support style demonstrated heightened autonomous motivation and more positive academic emotions. Furthermore, these positive effects were mediated by students’ autonomy need satisfaction. The fNIRS results revealed that, compared to the teachers’ control style, the students and teachers in the teachers’ autonomy support style exhibited enhanced interpersonal neural synchronization (INS) in the left prefrontal cortex (lPFC). This INS was positively associated with autonomy need satisfaction and positive emotions, with consistent findings observed in dynamic teacher–student INS. These findings provide a basis for further exploration into the neural mechanism underlying autonomy need satisfaction.

## Introduction

Self-determination theory (SDT) believes that psychological needs are essential for individual adaptation, integrity, and growth, serving as critical psychological nourishment (Deci and Ryan 2000). Humans have three fundamental psychological needs: autonomy, competence, and relatedness. Among them, autonomy is the most crucial, referring to a sense of willingness and psychological freedom (Van Assche et al. 2018). When individuals can make choices based on their own desires, their autonomy need is fulfilled. In recent years, SDT has been extensively applied in education, significantly influencing school education practices (Niemiec and Ryan 2009).

As key figures in education, teachers can create autonomy-supportive environment, satisfying students’ need for autonomy (Deci and Ryan 2000, Leenknecht et al. 2017). Teachers using this approach to respect students’ perspectives, engage in meaningful exchanges about learning value, respond to students’ viewpoints, and provide choice opportunities (Cheon et al. 2019, Jang and Reeve 2021). In contrast, controlling teachers ignore students’ views, force students to think or act in a particular way, use controlling language (e.g. you should, you must), and assign meaningless tasks (Reeve 2009, Soenens et al. 2012).

Many studies show that teacher autonomy support benefits students’ autonomous motivation, academic emotions, and achievement more than control style (Quested and Duda 2010, 2011, Jang et al. 2012, Delrue et al. 2019, Garn et al. 2019). However, most use questionnaires, with fewer using behavioral experiments that manipulate teacher styles via instruction priming (Grolnick and Ryan 1987, Benita et al. 2014) or comic situation substitution (Delrue et al. 2019, Baten et al. 2020). Regardless of method, the experiments often lack teacher–student interaction, reducing ecological validity. Comic situation substitution may reduce immersion and realistic responses. To better capture the real teaching complexity, this study should integrate both instruction priming with scenarios mimicking genuine teacher autonomy-supportive or controlling behaviors, enhancing ecological validity, ensuring that outcomes are more representative of true educational settings.

A substantial body of research, guided by SDT, has identified the specific teacher behaviors that support student autonomy (e.g. Tsai et al. 2008, Patall et al. 2017, Howard et al. 2025). Extensive evidence shows that these supportive behaviors are pivotal for students, satisfying their need for autonomy and enhancing academic performance (Quested and Duda 2010, Jang et al. 2012). Despite these well-documented behavioral patterns, the neural processes that enable a teacher to perceive a student’s perspective and translate it into supportive action—especially in real-time—remain poorly understood. Bridging this gap necessitates an investigation into the neural architecture of advanced social cognition. The prefrontal cortex (PFC) is a primary candidate, given its general involvement in teacher–student interactions (Holper et al. 2013, Takeuchi et al. 2016). More directly, Di Domenico et al. (2022) found that when a relationship is highly need-satisfying, the neural representation of a close other in the medial PFC (mPFC) overlaps with that of the self. Since autonomy support is a need-satisfying social context, this finding strongly implicates the PFC as a critical locus for its neural processing. Furthermore, providing autonomy support requires the teacher to accurately infer the student’s perspective, a mentalizing process consistently linked to the right temporoparietal junction (rTPJ) (Jiang et al. 2015, Brockington et al. 2018, Zheng et al. 2018). Based on this converging evidence, the PFC and rTPJ were selected as the primary regions of interest (ROI) for the present investigation. This study thus aimed to bridge educational psychology and neuroscience by uncovering the neural mechanisms that underlie the positive effects of teacher autonomy support.

Functional near-infrared spectroscopy (fNIRS) hyperscanning technology allows for the dynamic investigation of teacher–student interactions (Holper et al. 2013, Brockington et al. 2018, Pan et al. 2018, 2020, Zheng et al. 2018, Liu et al. 2019, Sun et al. 2020). Unlike traditional neuroimaging methods, fNIRS measures the brain activity of multiple individuals simultaneously with minimal interference from movement, which is essential for capturing real-life teaching scenarios (Montague et al. 2002, Baker et al. 2017, Balters et al. 2020). Interpersonal neural synchrony (INS) refers to the phenomenon in which the brain activity of two or more individuals becomes coupled or aligned while they engage in a shared activity or social interaction (Hasson et al. 2012). Recent studies suggest that such synchrony reflects a state of ‘resonance’ that signifies high-quality interaction and mutual engagement (Holper et al. 2013). In the classroom, teacher–student INS refers to this neural alignment during teaching interactions, which acts as an objective measure for the dynamic assessment of the interaction and overall teaching effectiveness (Liu et al. 2019). INS can predict learning outcomes and dynamically evaluate teaching quality (Holper et al. 2013, Brockington et al. 2018, Meshulam et al. 2021; for a review, see Tan et al. 2023). This neural coupling, or ‘resonance’, is thought to reflect the degree of shared attention, information transfer, and mutual understanding within the dyad’s interaction. Autonomy-supportive teaching is hypothesized to be particularly conducive to fostering this neural alignment. This teaching style—characterized by taking the student’s perspective, providing rationale, and offering choices—creates a collaborative environment that inherently demands deeper shared cognitive processing and mutual engagement compared to controlling teaching behaviors. Consequently, a positive association between autonomy-supportive teaching and teacher–student INS is predicted.

**Figure 1.**
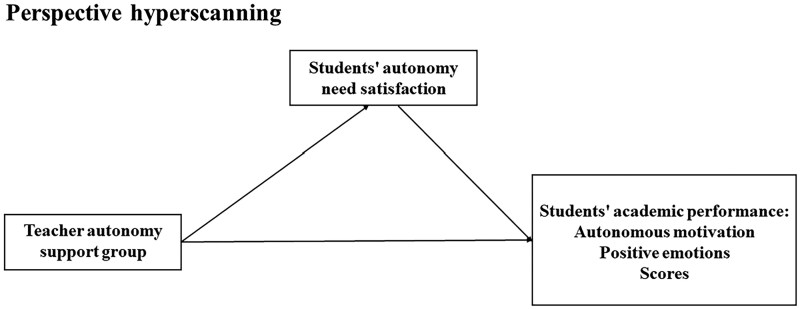
Hypothesized model.

Based on the SDT, this study utilized fNIRS hyperscanning to detect teacher–student INS in the PFC and rTPJ during a lab-simulated teaching process. This study aimed to provide neurobiological evidence on how teacher autonomy support affects students’ academic performance and its mechanisms. The study used a single-factor multivariate experimental design. The independent variable was the teacher autonomy support style, and its opposite, the teacher control style, was considered the control group. Students’ autonomy need satisfaction was the mediator, while autonomous motivation, academic emotions, and test scores were dependent variables. We hypothesized (see Fig. 1): (i) Students perform better with teacher autonomy support style than teacher control style. (ii) Teacher autonomy support would improve students’ performance via students’ autonomy need satisfaction. (iii) Teacher–student INS would be stronger under the teacher autonomy support style. (iv) INS would be positively correlated with autonomy need satisfaction, autonomous motivation, positive emotions, and score, and negatively with negative emotions.

## Methods

### Participants

This study was not preregistered. Twelve master’s students from a teacher education university received rigorous training: mastering the ‘Syllabus of Number Sequence Reasoning’, learning autonomy-supportive and control teaching styles, practicing each teaching style twice, and being evaluated for proficiency. Finally, four teachers (two males, two females) were selected based on student evaluations, ensuring their performance accurately matched the intended teaching style groups.

Sixty-four undergraduate liberal arts students unfamiliar with the test material were randomly assigned to receive either autonomy-supportive or control-style teaching from one of four teachers, forming 64 pairs. Each teacher taught 16 times (8 per style).

From the 64 datasets, 22 were excluded due to mismatched perceived and assigned styles (*n *= 10), non-serious responses (*n *= 1), lost NIRS markers (*n *= 1), and poor signal quality (*n *= 10; defined as a scalp coupling index below the threshold of 0.75, following the criterion established by Pollonini et al. (2014), leaving 42 valid datasets. These students included 11 males and 31 females, aged 18–23 years (*M *= 19.93, *SD *= 1.35).

To ensure that gender did not function as a confounding variable, we examined the gender composition of our final sample: 5 male-teacher–male-student, 13 male-teacher–female-student, 6 female-teacher–male-student, and 18 female-teacher–female-student. Pairs were categorized as ‘same-gender’ or ‘mixed-gender’. The autonomy support group had 11 same-gender pairs (3 male-teacher–male-student and 8 female-teacher–female-student) and 9 mixed-gender pairs, similar to the controlling group, which had 12 same-gender pairs and 10 mixed-gender pairs (*χ*^2^ = 0.001, *P *= .98). This result indicates that gender did not confound the manipulation.

### Materials and tasks

Following Zheng et al. (2018), teachers instructed students on sequence reasoning using materials from the ‘Chinese Civil Servants Administrative Professional Knowledge Level Tests’. A pre-experiment was conducted to finalize the test questions and to evaluate the effectiveness of teaching materials. Teachers adopted one-to-one teaching methods. To manipulate teacher autonomy-supportive and control styles, the study utilized instructional initiation techniques as outlined by Benita et al. (2014) and Grolnick and Ryan (1987), with specific style requirements from recent studies (Cheon et al. 2019, Jang and Reeve 2021), as detailed in Table 1.

**Table 1.** Manipulation of teacher autonomy support and teacher control style

	Teacher autonomy support group	Teacher control group
Instruction	The objective of teaching is to assist you in mastering sequence reasoning skills. While the task might seem challenging, it will foster your logical thinking. Any confusion during the learning process can be addressed at any time. Following the study period, a short assessment will evaluate your grasp of the material. It’s intended to gauge your learning progress, so there’s no reason to be excessively worried about the outcomes.	Today, what you have to do is to follow the teacher to grasp the knowledge of sequence reasoning. As long as you adhere to my line of reasoning, this task will not be difficult. During the learning process, you may only ask questions when permitted. In essence, you must stick with me to acquire this knowledge, as there will be a test afterwards to evaluate your understanding.
Requirements in the teaching process	1. Teachers will facilitate the exchange of learning materials with students, nurturing logical thinking skills. 2. Encourage the use of empowering language like ‘you can’ instead of demanding phrases such as ‘you must’. 3. From the students’ perspective, it’s important to honor their ideas. 4. In tackling complex sequence series, teachers will acknowledge the challenge and empathize with students’ frustrations. 5. Teachers will consistently exhibit patience while teaching and addressing students’ inquiries. 6. Offer students more autonomy by allowing them to choose their problem-solving methods, decide on the duration for solving problems, determine when to seek assistance from teachers, and choose to bypass certain topics they prefer.	1. Teachers neglect to elucidate the significance of learning materials, focusing instead on the tasks students are required to complete. 2. Use control languages such as ‘you should’ and ‘you must’. 3. Students are required to conform to the teachers’ methods of instruction, with their own ideas often overlooked. 4. Teachers perceive these questions as straightforward and believe learning them is simple, attempting to alter students’ negative feelings. 5. Teachers offer only cursory responses to students’ inquiries, displaying impatience when faced with numerous questions. 6. Students are denied the choice, with teachers dictating the timing for independent problem-solving, the duration, the methodology, and when inquiries can be made.

### Procedures

**Figure 2.**
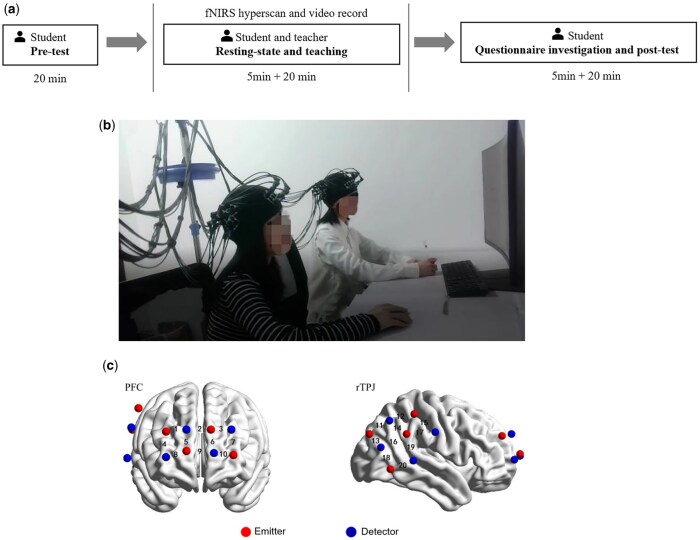
Experimental design. (a) Experimental procedures; (b) experimental paradigm; (c) Position of Optodes and channels.

The experimental process is shown in Fig. 2a. Students first completed a 20-minute pretest. Then, teachers and students wore fNIRS devices for a 5-min resting-state. The teaching process lasted about 20 min, with teachers and students positioned side by side facing a computer (Fig. 2b). Both sessions were video recorded. After teaching, students completed questionnaires on teacher autonomy support, motivation, emotion, and autonomy need satisfaction. Finally, students undertook a 20-min post-test.

### Behavioral data measurement

#### Teacher autonomy support

Students’ perceived teacher autonomy support was measured by the 6-item Learning Climate Questionnaire (Williams and Deci 1996), for example, ‘I feel that the teacher gives me the opportunity to choose’. Teacher control was measured using the 4-item Controlling Teacher Scale (Jang et al. 2009), e.g. ‘teachers use oppressive language, such as ‘must’.’ Both used 5-point Likert scales; higher scores indicate greater perceived support or control. Cronbach’s α were 0.96 (autonomy support) and 0.92 (control).

#### Autonomy need satisfaction

This was assessed with 4 items from the Basic Psychological Need Satisfaction scale (Chen et al. 2015), for example, ‘in this class, I can express my real thoughts at any time’, rated on a 5-point scale. Higher scores indicate greater autonomy satisfaction. Cronbach’s α was 0.93.

#### Autonomous motivation

Measured by Ryan and Connell (1989) Academic Self-Regulation Questionnaire with four dimensions: external, introjected, identity regulation, and intrinsic motivation. The Relative Autonomous Motivation (RAI) score was calculated as: RAI = (Intrinsic × 2) + Identity—Introjected - (External × 2). Ratings were on a 5-point scale; higher scores indicate higher autonomous motivation. Cronbach’s α were 0.64, 0.75, 0.85, and 0.84, respectively.

#### Academic emotions

The Academic Emotions Questionnaire (Pekrun et al. 2002) assessed eight emotions with one item each: pleasure, hope, pride (positive) and anger, anxiety, shame, helplessness, boredom (negative). Rated on a 9-point scale. Cronbach’s α were 0.83 (positive) and 0.76 (negative). An additional question (‘How is my emotional state during the learning process?’) assessed overall emotional state.

#### Test scores

The pre-test and post-test assessed the same content, including multiple series and power series, with both a medium level of difficulty as established by the pre-experiment. Each test comprised eight single-choice questions, for a maximum possible score of 8 points.

### fNIRS data acquisition

In this study, Shimadzu LABNIRS desktop near-infrared brain functional imaging system was used to collect brain signals at 66.667 Hz. 20 channels covered the PFC and rTPJ (Fig. 2c). During detection, the transmitter emits three wavelengths (780, 805, and 830 nm) of near-infrared semiconductor laser to the brain, and the detector receives light passing through the brain. The study measured PFC and rTPJ activity in teachers and students during rest and teaching, obtaining oxygenated hemoglobin, deoxyhemoglobin and total hemoglobin concentration changes in each channel.

The ROI in this study were the PFC and rTPJ. According to the Bruderman partition (Lancaster et al. 2000), channel 1, channel 4, channel 5, and channel 8 were regarded as the rPFC, channel 3, channel 6, channel 7, and channel 10 were regarded as the left prefrontal cortex (lPFC), channel 12, channel 14, channel 15, channel 16, channel 17, and channel 19 were regarded as the rTPJ, as shown in Table 2. To address potential validity concerns regarding moderate probabilistic coverage (e.g. CH19 at 49% for rTPJ), we applied two criteria: (1) the channel’s maximum coverage probability corresponded to our predefined ROI and (2) secondary regions exhibited lower probability. This approach aligns with previous studies (e.g. Leng et al. 2024, Song et al. 2024, Ming et al. 2025). Therefore, these 14 channels were included in the analysis, while the remaining 6 channels (2, 9, 11, 13, 18, 20) were excluded from analysis due to their location outside our predefined ROIs.

**Table 2.** Channel position

Channels	MNI coordinate			Bruderman partition,	
	*x*	*y*	*z*	Brain regions	Probability
CH1	23	60	33	9—Dorsolateral prefrontal cortex	.66
CH2	3	62	32	9—Dorsolateral prefrontal cortex	.63
CH3	−19	60	33	9—Dorsolateral prefrontal cortex	.65
CH4	35	62	20	10—Frontopolar area	.82
CH5	14	70	22	10—Frontopolar area	.84
CH6	−12	70	20	10—Frontopolar area	.82
CH7	−32	62	20	10—Frontopolar area	.83
CH8	23	72	9	10—Frontopolar area	.99
CH9	1	68	10	10—Frontopolar area	.99
CH10	−21	71	10	10—Frontopolar area	.98
CH11	47	−75	40	19—V3	.69
CH12	58	−51	51	40—Supramarginal gyrus part of Wernicke’s area	.65
CH13	49	−80	26	19—V3	.72
CH14	62	−55	40	40—Supramarginal gyrus part of Wernicke’s area	.57
CH15	67	−28	46	40—Supramarginal gyrus part of Wernicke’s area	.74
CH16	63	−60	26	39—Angular gyrus, part of Wernicke’s area	.61
CH17	70	−31	35	40—Supramarginal gyrus part of Wernicke’s area	.80
CH18	60	−68	7	19—V3	.56
CH19	70	−42	19	40—Supramarginal gyrus part of Wernicke’s area	.49
CH20	69	−51	0	37—Fusiform gyrus	.34

### Data analysis

#### Behavioral data analysis

First, the independent sample *t*-test was used to analyze differences in students’ autonomy need satisfaction, autonomous motivation, and academic emotions, while two-factor repeated measurement analysis of variance was used to test differences in test scores. Then, the bias-corrected percentile Bootstrap method (5000 times) was used to test mediation models.

#### fNIRS data analysis

Matlab R2013b software with spm8, NIRS_SPM, wavelet-coherence, and BrainNetViewer were used. First, the data were preprocessed to mitigate motion artifacts using a wavelet-based filtering method (Brigadoi et al. 2014), which employed a Daubechies 5 wavelet and a parameter of *α *= 0.1 (Molavi and Dumont 2012;, Brigadoi et al. 2014). Subsequently, the processed optical signal was converted into blood oxygen data using a modified Beer-Lambert law, focusing solely on the concentration change of oxygenated hemoglobin due to its sensitivity to the task (Doi et al. 2013). Second, the resting phase retained continuous 3-min baseline data (minutes 2–4 of the 5-minute resting phase), excluding only the initial and final 60 s (Zhu 2020). During teaching, a shared time period of approximately 14.6 min for all pairs of subjects was used. ROI analysis averaged blood oxygen data from multiple channels within each ROI before analysis (Dai et al. 2018).

Wavelet transform coherence analysis was used to analyze teacher-student blood oxygen correlations in three brain regions (Grinsted et al. 2004). Before the statistical test, the data needed to be averaged in time and frequency. To ensure that the selected frequency band was related to the teaching task, the coherence value of each frequency in 0.01–0.2 Hz was averaged in time. This range was chosen to remove the influence of physiological signals, such as cardiac pulsation (about 0.7–4 Hz), respiration (about 0.2–0.3 Hz) (Xue et al. 2018), while encompassing the frequency bands concerned by many interpersonal interaction studies (Pan et al. 2018, Sun et al. 2020). Then, all the pairs of teachers and students matched were randomly disrupted, and the task-related INS was defined as the INS difference of the coherence value from real subject pairs relative to the disrupted subject pairs. The task-related INS was converted into *z*-scores using Fisher z-statistics. A series of independent sample *t*-tests was performed on the INS for each brain region in each frequency in the two groups, *P *< .05. Since this was only used to determine the task-related frequency band, rather than to obtain the result, no further correction of the multiple comparisons was performed (Zheng et al. 2018).

**Figure 3.**
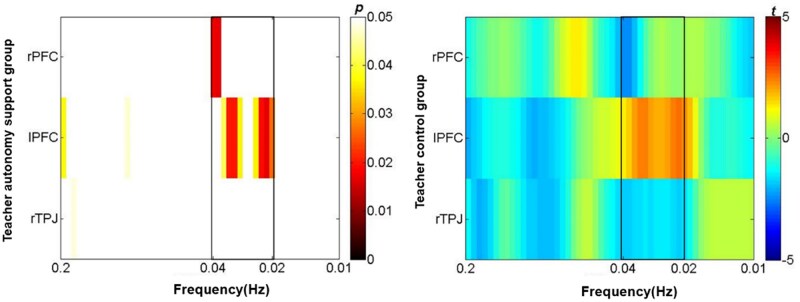
The P-value diagram and *t*-value diagram of the INS difference test between the teacher autonomy support group and the teacher control group during the 0.01–0.2 Hz frequency band.

Figure 3 shows teacher autonomy support group had higher INS at 0.02–0.034 Hz, control group had higher INS at 0.036–0.04 Hz, leading us to select 0.02–0.04 Hz as the task-related frequency band. Within this band, frequencies were averaged. We then used FDR-corrected one-sample *t*-tests to confirm INS > 0 and independent sample *t*-tests to compare groups across brain regions.

To examine the earliest time point at which the INS difference between teacher autonomy support and teacher control style appeared, time course analysis was used to understand the dynamic INS changes in the teaching process (Liu et al. 2019, Liang et al. 2022). When analyzing the time course of fNIRS data, commutative data can provide more stable prediction accuracy than instantaneous data (Jiang et al. 2015). First, as with the analytical methods used earlier in this study, 14.6 minutes (878 s) of teaching stage for all pairs of subjects were used in time course analysis. Each second was seen as an epoch. Second, the cumulative INS over time for each dyad at epoch *n* was determined by aggregating INS values sequentially from the initial epoch through the *n*th observation period. The temporal accumulation of INS in the significant brain regions in the wavelet coherence analysis was performed in the teacher autonomy support group and the teacher control group, respectively. Third, the time-cumulative INS at each time point was compared between the groups to examine the dynamic changes in INS during the teaching process.

#### Relationship between behavior and INS

Pearson correlation was used to analyze the correlation between INS and behavioral results, as well as the association between the cumulative INS at each time point during the teaching process and behavioral outcomes (Liang et al. 2022).

## Results

### Manipulation check

Students in the teacher autonomy support group perceived significantly higher teacher autonomy support, *t*(19) = 16.902, *P *< .001, while the teacher control group perceived significantly higher teacher control, *t*(21) = 9.673, *P *< .001. These findings confirm the manipulation.

### Background variables

Results showed no significant difference in background variables between the two groups (gender ratio: *χ*^2^ = 0.29, *P *= .592; grade ratio: *Z* = −0.37, *P *= .715; age: *t*_(40)_= −0.14, *P *= .890; pre-test scores: *t*_(40)_ = 0.52, *P *= .609; matching teachers ratio: *P *= .258; teaching time: *t*_(40)_ = −0.52, *P *= .606). These results indicate that these variables will not interfere with the study’s findings.

### Behavioral results

**Figure 4.**
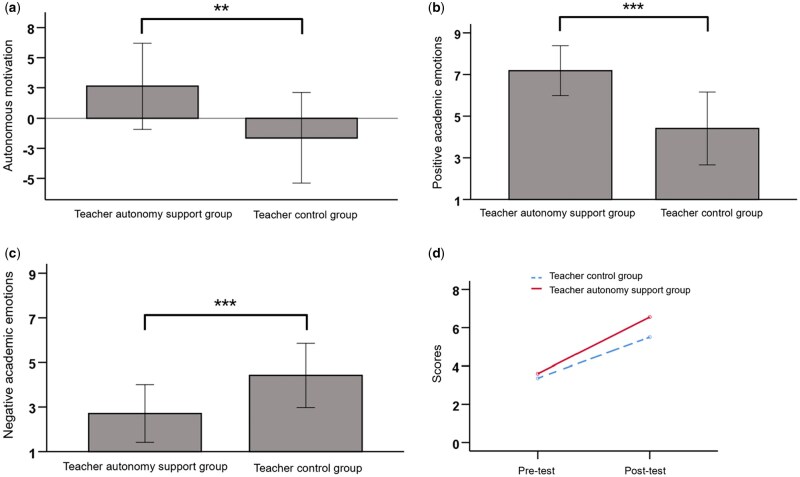
The influence of teacher autonomy support on students’ academic performance. (a) Autonomous motivation; (b) positive academic emotions; (c) negative academic emotions; (d) Scores. Error bars represent standard deviation.

Descriptive statistics are in Table 3. Compared to the control group, the teacher autonomy support group had higher autonomy need satisfaction (*t*_(40)_ = 13.70, *P *< .001, *d *= 4.20), RAI (*t*_(40)_ = 3.78, *P *= .001, *d *= 1.17, Fig. 4a) and positive emotions (*t*_(40)_=5.94, *P *< .001, *d *= 1.85; Fig. 4b), with lower negative emotions (*t*_(40)_ = −4.03, *P *< .001, *d* = −1.25; Fig. 4c).

**Table 3.** Descriptive statistics on behavioral variables (*M*±*SD*)

Variables	Teacher autonomy support group (*n *= 20)	Teacher control group (*n *= 22)
Autonomy need satisfaction	3.97 ± 0.68	1.51 ± 0.48
RAI	2.64 ± 3.56	−1.64 ± 3.75
Positive emotions	7.18 ± 1.20	4.41 ± 1.75
Negative emotions	2.71 ± 1.29	4.42 ± 1.44
Pre-test scores	3.60 ± 1.64	3.36 ± 1.33
Post-test scores	6.55 ± 1.57	5.50 ± 2.24

A two-factor repeated ANOVA revealed that a significant main effect of time of test (*F*_(1,40)_ = 57.20, *P *< .001, *η*^2^  *partial *= 0.59; Fig. 4d). However, there was no significant effect for group (*F*_(1,40)_ = 2.38, *P *= .131) or the group × time interaction (*F*_(1,40)_ = 1.46, *P *= .233).

In addition, two graduate students majoring in psychology encoded open questions of academic emotions into positive and negative categories. A total of 9 data were missing and 33 valid data were left. The results showed that the Kappa value was 0.934 (*P *< .001), indicating good rater consistency. Disagreements were resolved through discussion (Table 4).

**Table 4.** The number of students’ academic emotions in teacher autonomy support group and teacher control group

	Teacher autonomy support group (*n *= 16)	Teacher control group (*n *= 17)	Total (*n *= 33)
Positive academic emotions	15	3	18
Negative academic emotions	1	14	15

For the binary logistic regression, groups (control = 0, support = 1) and emotions (negative = 0, positive = 1) were coded. Results showed that students in the teacher autonomy support group had 70 times higher odds of positive emotions than the control group (*b *= 4.25, Wald = 12.27, *P *< .001, 95% OR 6.50–754.44).

To test whether autonomy need satisfaction plays a mediating role, correlation analysis was performed between group, RAI, academic emotions, autonomy need satisfaction, and scores. As shown in Table 5, based on correlations, the mediating effect of autonomy need satisfaction between teacher autonomy support group and autonomous motivation, positive emotions, and negative emotions would be tested.

**Table 5.** Bivariate correlations between variables (*N *= 42)

	1	2	3	4	5	6	7
1 Groups	–						
2 RAI	0.51^***^	–					
	0.26–0.74						
3 Positive academic emotions	0.69^***^	0.73^***^	–				
	0.52, 0.82	0.54, 0.88					
4 Negative academic emotions	−0.54^***^	−0.69^***^	−0.61^***^	–			
	−0.74, −0.30	−0.81, −0.52	−0.77, −0.40				
5 Autonomy need satisfaction	0.91^***^	0.67^***^	0.81^***^	−0.58^***^	–		
	0.88, 0.94	0.47, 0.82	0.70, 0.90	−0.76, −0.34			
6 Post-test scores	0.27	0.46^**^	0.43^**^	−0.28	0.33^*^	–	
	−0.02, 0.49	0.20, 0.65	0.20, 0.61	−0.56, 0.09	0.04, 0.54		
7 △ scores	0.19	0.31^*^	0.26	−0.11	0.15	0.76^***^	–
	−0.11, 0.45	0.03, 0.54	−0.09, 0.55	−0.40, 0.24	−0.19, 0.44	0.54, 0.88	
8 INS at lPFC	0.40^**^	0.23	0.36^*^	−**0.29**	0.40^**^	0.25	0.03
	0.14, 0.62	−0.04, 0.50	0.02, 0.63	−0.52, −0.02	0.14, 0.63	−0.01, 0.46	−0.29, 0.36

Note: the difference between the post-test scores and the pre-test scores is regarded as the Δ scores. Bold indicates that *P *> .05, but the confidence interval obtained by bootstrap sampling for 5000 times does not contain 0.

**Figure 5.**
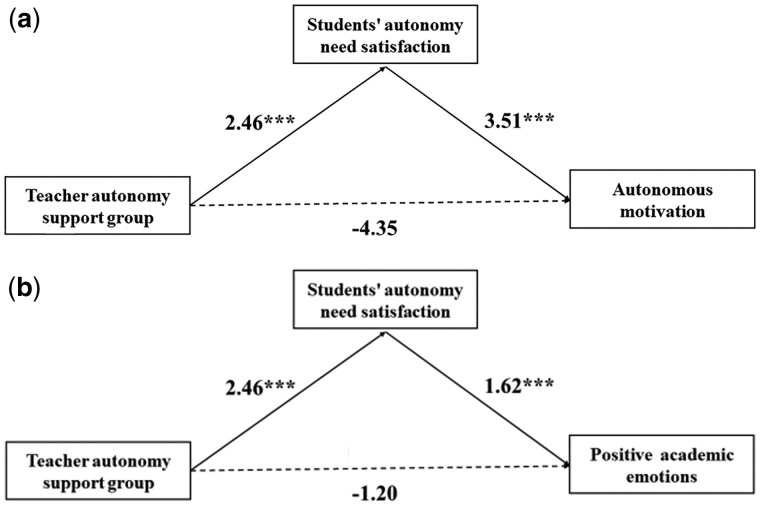
The mediating effect diagrams. (a) The mediating role of autonomy need satisfaction between autonomy support group and autonomous motivation; (b) The mediating role of autonomy need satisfaction between autonomy support group and positive academic emotions.

First, the mediating effect of autonomy need satisfaction on the relationship between teachers’ autonomy support group and students’ autonomous motivation was 8.63, 95% CI 4.58–13.35, indicating that the mediating effect was significant. The direct effect was not significant (direct effect = −4.35, 95% CI −9.63, 0.25). Therefore, autonomy need satisfaction played a full mediating role, as shown in Fig. 5a.

Second, the mediating effect of students’ autonomy need satisfaction on positive emotions was significant (indirect effect = 3.97, 95% CI 2.63–5.64). The direct effect was not significant (direct effect = −1.20, 95% CI −2.88, 0.23). Therefore, autonomy need satisfaction played a full mediating role, as shown in Fig. 5b.

Finally, the mediating effect of autonomy need satisfaction on negative emotions was not significant (indirect effect = −1.46, 95% CI −3.36, 0.33). Therefore, autonomy need satisfaction did not play a mediating role.

### fNIRS results

Descriptive statistics are in Table 6. First, one-sample *t*-tests showed that, in the teacher autonomy support group, task-related INS was found in the lPFC, *t* (19) = 3.44, *P *= .0027, *d *= 1.58, FDR corrected. In the teacher control group, there was no significant task-related INS. Second, in the lPFC, task-related INS in the teacher autonomy support group was significantly higher than the teacher control group, *t* (40) = 2.78, *P *= .0082, *d *= 0.86, FDR corrected.

**Table 6.** Descriptive statistics on task-related INS (*M*±*SD*)

	Teacher autonomy support group (*n *= 20)	Teacher control group (*n *= 22)
lPFC	0.0585 ± 0.0761	−0.0115 ± 0.0859
rPFC	0.0006 ± 0.0593	0.0164 ± 0.0812
rTPJ	−0.0114 ± 0.0807	0.0404 ± 0.0861

**Figure 6.**
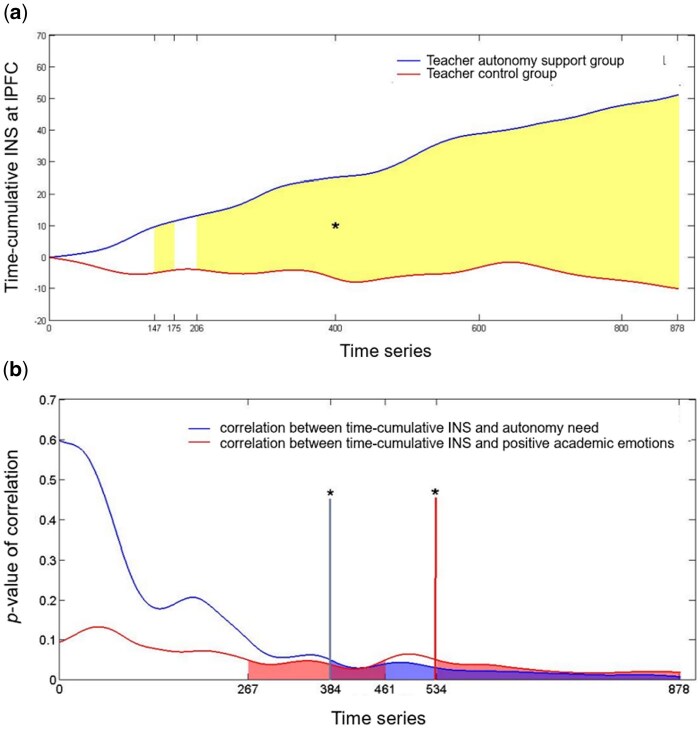
Dynamic changes of time-cumulative INS. (a) The difference test between the teacher autonomy support group and the teacher control group on the time-cumulative INS; (b) The *P*-value diagram of the correlation between time-cumulative INS and students’ autonomy need, between time-cumulative INS and positive academic emotions.

Time course analysis showed that, from the 206th second, time-cumulative INS significantly increased in the teacher autonomy support group compared to the teacher control group, *P *< .05 (Fig. 6a).

### Correlations between behavioral results and fNIRS results

**Figure 7.**
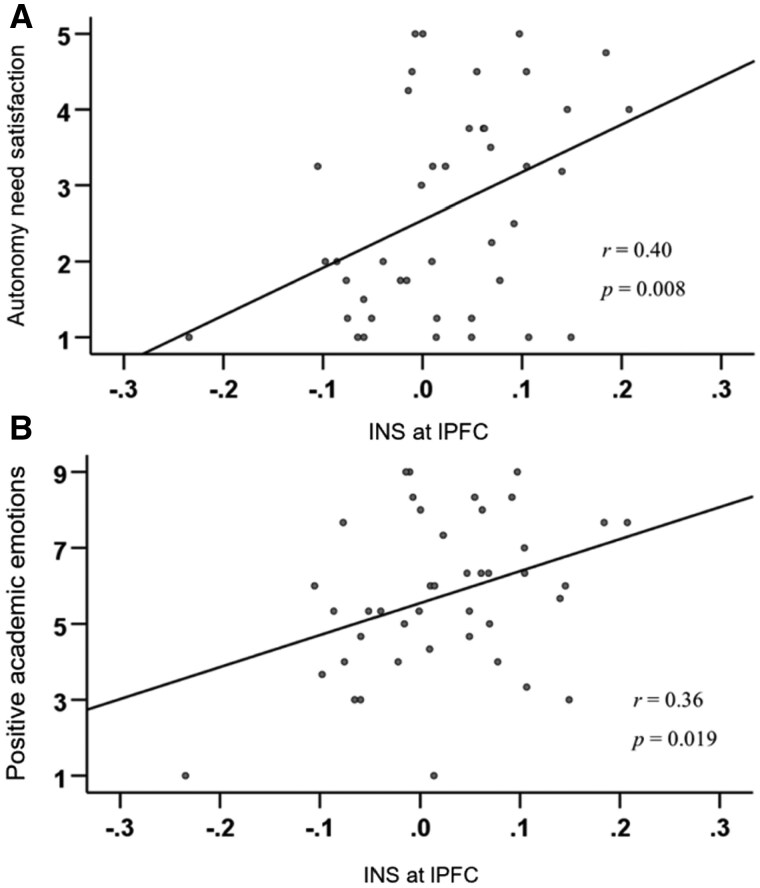
Scatter diagrams. (a) The correlation between INS and students’ autonomy need satisfaction; (b) The correlation between INS and students’ positive academic emotions.

The INS in lPFC was positively correlated with group (*r *= 0.40, *P *= .008), autonomy need satisfaction (*r *= 0.40, *P *= .008; Fig. 7a), and positive emotions (*r *= 0.36, *P *= .019; Fig. 7b). Similarly, time-cumulative INS in lPFC was positively correlated with autonomy need satisfaction from the 384th second (*P *< .05), and positive emotions from the 534th second (*P *< 0.05; Fig. 6b). For both measures, there were no correlations with other behavioral results.

## Discussion

The study used fNIRS hyperscanning and questionnaires to investigate the impact of teacher autonomy support on students’ autonomous motivation, emotions, and performance. Students in the autonomy support group exhibited heightened autonomous motivation and more positive emotions, mediated by autonomy need satisfaction. fNIRS results revealed increased lPFC activation in autonomy support group, which correlated with autonomy need satisfaction and positive emotions, confirmed by time course analysis.

### The influence of teacher autonomy support on students’ academic performance and its mechanism

Students experiencing teacher autonomy support showed stronger autonomous motivation and more positive academic emotions. This indicates that when teachers offer autonomy support, students are more willing to explore and tackle challenges. These findings support the hypothesis and confirm previous studies (Quested and Duda 2010, Garn et al. 2019).

However, the autonomy-supportive teaching style failed to yield significantly higher test scores compared to the control condition, a finding inconsistent with our initial hypothesis. We propose a twofold explanation for this null result. First, consistent with SDT, the impact of autonomy-supportive teaching on academic achievement is indirect, mediated by more proximal outcomes like the satisfaction of basic psychological needs and academic motivation (Ryan and Deci 2020). Meta-analyses show that while autonomy support has medium-to-large effects on need satisfaction and autonomous motivation (*r* = 0.27 to 0.62; Bureau et al. 2022), its effect on academic achievement is consistently smaller (e.g. *r* = .16; Tao et al. 2022). Given this modest effect size, our single-session teaching was not long or intense enough to produce a statistically detectable change in post-test scores. Second, a potential ceiling effect may have contributed. The teaching materials were logically structured and straightforward, which created a ceiling effect, allowing students in both conditions to perform at a high level and thus masking the potential benefits of the autonomy-supportive style.

Students’ autonomy need satisfaction fully mediated the relationship between teacher autonomy support and students’ autonomous motivation and positive academic emotions. These findings align with previous studies (Quested and Duda 2010). Chen et al. (2015) found that autonomy need satisfaction fully mediated the link between autonomy support and motivation internalization, while competence and relatedness needs only partially mediated it. Therefore, our findings confirm the importance of autonomy need satisfaction in academics.

### The influence of teacher autonomy support on teacher–student INS

As hypothesized, teachers and students in the teacher autonomy support group exhibited higher INS in the lPFC than the control group. The teacher control group showed no significant INS differences between real and disrupted pairs, implying ineffective communication. The teacher–student INS detected in the lPFC aligns with previous studies (Takeuchi et al. 2016, Zheng et al. 2018, Liu et al. 2019), as the lPFC is related to positive emotions (Wheeler et al. 1993) and of self-other information integration (Takeuchi et al. 2016). During teaching, both teachers and students integrate self-referential and other-referential information, which INS in the lPFC may reflect.

However, teacher–student INS was absent in the rTPJ, consistent with previous teaching hyperscanning studies (Jiang et al. 2015, Pan et al. 2018, Liu et al. 2019), which found INS in frontal regions like the lPFC and subfrontal cortex. Liu et al. (2019) suggested the TPJ and PFC may form a network during social learning, but further studies are needed to clarify their roles.

### The relationship between teacher–student INS and behavioral results

In line with our hypothesis, teacher–student INS in the lPFC was positively associated with students’ autonomy need satisfaction and positive emotions. This association may be linked to the lPFC’s role in processing positive emotions (Wheeler et al. 1993), transposition thinking, and inferring others’ intentions (Sun et al. 2020). The teacher autonomy support style embodies transposition thinking, potentially explaining the positive correlation between autonomy need satisfaction and INS in the lPFC.

A surprising yet informative finding was the lack of a significant correlation between INS and post-test scores, a result that diverges from prior studies (e.g. Zheng et al. 2018, Liu et al. 2019), which can be interpreted through two complementary explanations. First, this finding is attributed to the interaction design. A meta-analysis by Zhang et al. (2022) revealed that the mode of interaction is a powerful moderator of the link between INS and learning outcomes. A positive correlation between INS and learning outcome was found, but only in studies employing constant face-to-face interaction (*r *= 0.455). A plausible reason for this strong effect is that the face-to-face condition facilitates the rich integration of multi-modal social cues, including online speech, facial expressions, gestures, and eye contact. In contrast, for non-face-to-face interactions where such cues are limited or absent, the relationship was effectively null (*r* = −0.042). In our study, participants frequently focused on a shared screen rather than maintaining constant eye contact. Non-significant finding is therefore consistent with the meta-analytic evidence. Second, INS more closely reflects the quality of engagement and interpersonal ‘resonance’ (Holper et al. 2013, Tan et al. 2023) rather than the final test score. This was compounded by a probable ceiling effect on test performance, which limited variance and attenuated any potential brain–behavior correlation. Future studies should employ a face-to-face paradigm and more challenging post-tests.

### Dynamic teacher–student INS in the teaching process

#### Early identification of teacher autonomy support and teacher control styles through INS

At the 206th second, teacher autonomy support led to significantly higher time-cumulative teacher–student INS in the lPFC compared to the teacher control style. This trend persisted, indicating that teacher autonomy support fosters effective communication from early stages of instruction.

This observation deviates from previous studies, which found INS differences related to teaching methods at the 76th second (Zheng et al. 2018) or communication method (Liu et al. 2019) much earlier (around 65–90 s). In contrast, our study identified significant differences at 206 s, likely because teaching style emerges more gradually than teaching or communication methods. Given our study’s 878-second duration, 206 s is still within the early stage, allowing INS to differentiate these styles.

#### INS in the middle period can indicate students’ autonomy need satisfaction and positive emotions

The behavioral results of this study indicate that teacher autonomy support (as opposed to teacher control) leads to greater autonomy satisfaction and more positive academic emotions. This is further evidenced by the correlation between time-cumulative INS and autonomy satisfaction, as well as positive academic emotion. Beginning in the middle stage of teaching, time-cumulative lPFC INS showed significant positive correlations with students’ autonomy need satisfaction (from 384 s) and positive emotions (from 534 s), with both correlations persisting until the end. This confirms INS is a process variable, similar to these psychological outcomes, and fills a research gap regarding the relationship between time-cumulative INS and these subjective experiences. The dynamic interaction of time-accumulated INS and students’ academic performance suggests that teacher autonomy support is not just about the scores but is more deeply connected to the process (i.e. how the students feel and engage emotionally during learning). The findings emphasize that the INS between teacher and student during the learning process offers a more immediate reflection of teaching dynamics than scores do, offering new insights into how teaching styles impact learning at a deeper, neural level.

Contrary to previous studies, our research found no consistent correlation between time-cumulative INS and student test scores. Zheng et al. (2018) and Liu et al. (2019) reported positive correlations in different brain regions and times. Despite using similar materials to Zheng et al. (2018), our study differed, possibly due to brain regions differences. Zheng et al. (2018) found that INS increases in teachers’ anterior temporal cortex and students’ TPJ correlated with scores, but not INS in teachers’ TPJ and students’ TPJ. As they noted, ‘Different types of INS may mark different aspects of teacher-student interaction.’ In our study, time-cumulative INS in teachers’ and students’ lPFC can indicate students’ autonomy need satisfaction and positive emotions during interaction.

Our findings establish INS in the lPFC as a key neural marker that distinguishes between autonomy-supportive and controlling teaching styles but also signifies its connection to the student’s subjective experience. The heightened synchrony observed during autonomy–supportive interactions reflects enhanced shared attention, cognitive engagement, and emotional attunement (Koike et al. 2015, Zheng et al. 2020, Zhang et al. 2024). This lPFC INS is positively correlated with process-based outcomes, including students’ autonomy need satisfaction and positive affect, providing evidence for an ‘outside-in’ (teacher support shaping student brain activity) and ‘hinside-out’ (neural synchrony predicting well-being). These results lend neurophysiological support to a core pathway of Self-Determination Theory: the social context enhances motivation by nurturing psychological needs (Ryan and Deci, 2017). Practically, as portable technologies like fNIRS and EEG advance, INS is emerging as a viable ‘online’ marker for learning quality, unlike traditional interventions based on delayed cues. This would empower educators to dynamically adapt their teaching strategies—such as increasing autonomy support—when a dip in neural alignment is detected, paving the way for responsive and personalized neurofeedback-guided teaching.

### Limitations and prospects

This study has several limitations. First, fNIRS detects only surface cortical activity, missing deeper brain regions (e.g. ventromedial prefrontal cortex, amygdala, and striatum). Additionally, limited probes restricted the coverage to PFC and rTPJ. Future research should explore broader brain areas. Second, the lab simulation with one-to-one, short (20 min) teaching limits external validity; future work could use one-to-many setups or real classrooms. Third, using pre-service teachers and liberal arts students may not reflect the experienced teachers or diverse student populations. Future studies should expand these samples. Fourth, the task focuses on moderately difficult sequence reasoning, and other subjects and task difficulties should be assessed. Fifth, we did not classify teaching styles using neural data. This is promising for future research, since Pan et al. (2020) found that brain-to-brain coupling better classifies instruction than individual brain activity. Therefore, a compelling next step is to use machine learning on inter-brain coupling patterns. This approach could lead to an objective classification of teaching styles from neural interactions, offering a powerful tool for educational research and teacher development.

## Conclusion

This study found that compared to the teacher control style, the teacher’s autonomy support style is more conducive to students’ autonomous motivation, positive emotions, and communication. The INS in the lPFC serves as a neural marker of the differences between these two styles. Furthermore, teacher autonomy support enhances students’ autonomous motivation and promotes positive emotions by satisfying students’ autonomy needs. The INS in the lPFC is positively correlated with students’ autonomy need satisfaction and positive emotions, and consistent results were found in dynamic INS. Additionally, the mid-term INS can predict students’ autonomy need satisfaction and positive emotions, providing a starting point for further research on the neural mechanisms of autonomy need satisfaction.

## Data Availability

The data underlying this article will be shared on reasonable request to the corresponding author.
